# Bullying as a Group Process in Childhood: A Longitudinal Social Network Analysis

**DOI:** 10.1111/cdev.13298

**Published:** 2019-08-19

**Authors:** J. Ashwin Rambaran, Jan Kornelis Dijkstra, René Veenstra

**Affiliations:** ^1^ University of Groningen

## Abstract

This study investigates the dynamic interplay between bullying relationships and friendships in a sample of 481 students in 19 elementary school classrooms (age 8–12 years; 50% boys). Based on a relational framework, it is to be expected that friendships would be formed when two children bullied the same person and that children would start to bully the victims of their friends. Similarly, it is to be expected that friendships would be formed when two children were victimized by the same bully and that children would become victimized by the bullies of their friends. Longitudinal bivariate social network analysis supported the first two hypotheses but not the latter two. This study provides evidence for group processes in bullying networks in childhood.

School bullying affects the lives of many children. Victims of school bullying often experience weak social support, rejection, and social isolation that can be detrimental to their socioemotional development, such as weak social bonding, poor academic performance, and elevated levels of anxiety and depression (Kochenderfer & Ladd, 1996; Rivara & Le Menestrel, 2016). Children who transgress toward peers by bullying, in contrast, often experience support from peers, for example, by peers who assist in harassing the victim (Salmivalli, 2010). The negative consequences for those who bully others have also been well‐documented, such as low academic functioning in school to more serious offending later in life (Rivara & Le Menestrel, 2016).

The social components of bullying suggest that it is a group process (Salmivalli, 2010). This idea stems from the participant roles approach (Salmivalli, Lagerspetz, Björkqvist, Österman, & Kaukiainen, 1996), which emphasizes the social nature of bullying. Bullying often takes place at school, where victims are unable to avoid their bullies. Victims are often harassed by multiple bullies, and because of fear of rejection, only a few may defend the victim. Peer group members tend to have similar involvement in aggressive and bullying behaviors (Espelage, Green, & Wasserman, 2007; Espelage, Holt, & Henkel, 2003; Haselager, Hartup, van Lieshout, & Riksen‐Walraven, 2008; Salmivalli, Huttunen, & Lagerspetz, 1997). Similarity between friends in aggression and bullying may be driven by selection, referring to that aggressive children want to affiliate with each other (Cairns & Cairns, 1994), or by influence, referring to that friends become increasingly more similar in their behaviors over time (Guerra, Williams, & Sadek, 2011; Poulin & Boivin, 2000; Salmivalli & Voeten, 2004; Werner & Hill, 2010). Previous studies on dynamics in networks and behavior found no clear evidence that bullies select each other as friends or that bullies influence their friends in adolescence (Caravita, Sijtsema, Rambaran, & Gini, 2014; Merrin et al., 2018; Sentse, Kiuru, Veenstra, & Salmivalli, 2014; Sijtsema, Rambaran, Caravita, & Gini, 2014).

An explanation for the lack of support is that these previous network studies examined bullying as individual behavior. However, researchers have come to recognize that bullying is relational and that we should examine *who bullies whom*. From this relational or status framework (Rodkin, Espelage, & Hanish, 2015; Veenstra et al., 2007), it can be argued that bullies choose their friends and victims strategically. Aside from social affection, bullies also strive for social status (Salmivalli, 2010; Veenstra, Lindenberg, Munniksma, & Dijkstra, 2010). By targeting the weaker individuals in the group, they are able to create a power imbalance with their victims without losing affection from peers (Salmivalli, 2010; Veenstra et al., 2010). In order to maintain a high social status, friends may serve as social support during bullying incidents as well as help against defenders of victims. In line with this idea, recent empirical work on the interplay between positive and negative relationships showed that children who bullied the same person tend to defend each other (Huitsing, Snijders, van Duijn, & Veenstra, 2014). Other work also showed that adolescents who are befriended dislike the same persons (Rambaran, Dijkstra, Munniksma, & Cillessen, 2015). These studies also suggest that sharing the same victims, dislikes or enemies foster positive affect between individuals and increases the motivation to befriend each other. We build on these previous network studies by investigating the longitudinal interplay between friendships and bullying relationships in middle childhood to early adolescence.

## Background

Empirical research on bullying dates back to the 1970s in Scandinavia (for a brief review, see Hymel & Swearer, 2015), where it was labeled “mobbing,” referring to “school children repeatedly ganging up on the same victims” (Lagerspetz, Björqvist, Berts, & King, 1982, p. 45; Olweus, 1978). It was argued that bullying typically involves the same individuals, bullies, who single‐out others as targets of victimization. Bullies were found to be not only physically stronger than their victims (portraying a power imbalance) but also better adapted in the group (e.g., more popular). Although researchers already spoke of a group process in explaining bullying, the emphasis was put on specific bullies and specific victims.

The participant roles approach extends this line of research by considering that peer witnesses are present when bullying takes place and that it matters how these so‐called bystanders react to the bullying (Salmivalli et al., 1996; for a review, see Salmivalli, 2010). It was demonstrated that children have different roles in the bullying process. In addition to being bullies and victims, children can be assistants or reinforcers of bullies (e.g., by joining in or making fun of victims), outsiders (e.g., those who withdraw), or defenders of victims (e.g., those who help or comfort them). Children who value social status might select into bullying groups, by joining in, as a way to enhance their own social standing in the group (Witvliet et al., 2010). Within bully cliques, children might reinforce each other’s bullying behaviors by providing positive feedback through verbal and nonverbal cues (e.g., smiling or laughing), which might be socially rewarding (Salmivalli, 2010). This process is a form of peer influence, referring to bullies influencing their friends.

Middle childhood to early adolescence is an important period to examine selection and influence in bullying behaviors because children attach great value to developing positive relationships with peers during this time (Poulin & Chan, 2010). Friendships may provide children companionship, intimacy, loyalty, and affection, and influence their behaviors. Friendships, however, are moderately stable as children regularly lose old friendships while forming new ones. In the period from middle childhood to early adolescence, children become also aware of their own and others’ positions and roles in the group (Kolbert & Crothers, 2003). During this time, these positions and roles are moderately stable (Salmivalli, Lappalainen, & Lagerspetz, 1998) and many children switch bullying roles: They may be a bully at one time point, a victim at another, and uninvolved at the next time point (Zych et al., 2019). Stability and change in friendships are also linked to involvement in bullying (Poulin & Chan, 2010). Victims often maintain friendships with other victims but experience difficulties forming friendships with nonvictims. Aggressive children do not have a hard time making new friends with (non)aggressive children but have difficulties keeping their friends (Ellis & Zarbatany, 2007). These change and stability processes enable us to assess peer selection and influence in bullying behaviors.

## Hypotheses

We examine peer selection and influence processes in specific targets instead of general behavior. Thus far, only a few studies have used this dyadic approach in longitudinal social network research (e.g., Huitsing et al., 2014; Rambaran et al., 2015). These studies provided insight into the interplay between positive and negative networks, and revealed the relational aspects of bullying, disliking, and defending. For instance, a longitudinal study on the interplay between defending relationships and victimization relationships found, among a sample of children in late elementary school, that victims defended each other when they were being harassed by the same bully; the same was true for bullies with the same victims (Huitsing et al., 2014). Another longitudinal study on the interplay between friendships and antipathies, in which study participants were adolescents, found that friendships were formed when students disliked the same person and that students agreed with their friends on whom to befriend and dislike but disagreed with their antipathies (Rambaran et al., 2015). We continue this line of research by examining the interplay between friendships and bullying relationships in childhood. In what follows, we present four hypothetical configurations to clarify how friendship selection and influence processes may operate in bullying and victimization relationships.

### Bully Selection and Influence Hypotheses

**Figure 1:**
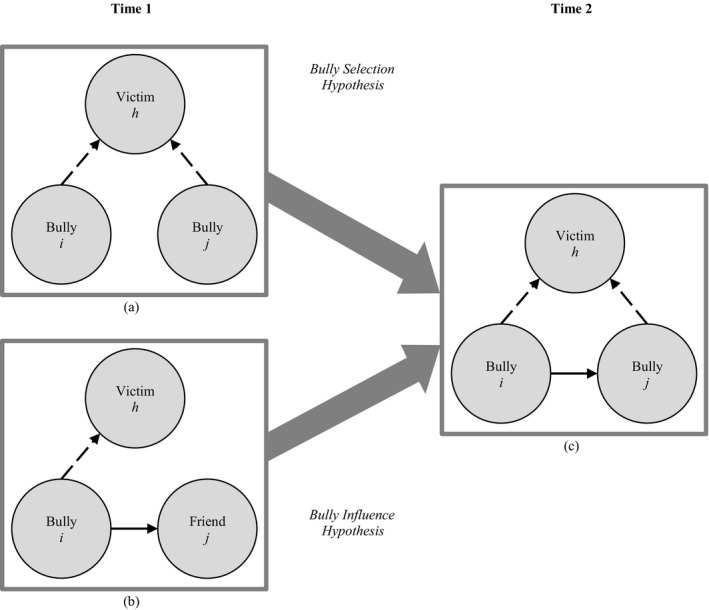
Illustration of the interplay between bullying (“Who do you bully?”) and friendship. As can be seen, the same outcome (c) can be produced by different underlying processes. By (a) two children who bully the same victim at Time 1 become friends at Time 2 (describing a selection process), and (b) a person who is friends with a bully at Time 1 starts to bully the same victim at Time 2 (describing an influence process). To facilitate the interpretation of these network configurations, friendships are represented with solid lines and bullying relationships are represented with dashed lines. This figure is derived from Huitsing et al. (2014).

The first configuration describes a situation where two individuals share the same victim, referring to that they bully the same person at the same time (Figure 1: a → c). In this configuration, it is expected that the first person’s relationship with the second becomes positive over time (referring to that *i* bullies *h* and *j* bullies *h*, then *i* likes *j*). In other words, when two individuals bully the same person they become friends. We call this the *bully selection* hypothesis, indicating that bullies select each other as friends. This selection effect may be explained by that similarity between two individuals breeds attraction (Byrne, 1971; Byrne & Nelson, 1965), which may provide social support and behavioral confirmation to the bullies. An alliance also secures one’s position in the group and offers protection against threats (Huitsing et al., 2014; Sainio, Veenstra, Huitsing, & Salmivalli, 2011). Bullies may protect themselves against potential retaliation from their victims or classmates who defend victims (Huitsing et al., 2014). Hence, bullies with the same victim may form alliances through friendships to obtain a strong position in the group, which may discourage others to side with the victim because of the fear to become victims themselves (Pozzoli & Gini, 2010).

The second configuration describes a situation where two individuals are friends, and, at the same time, one of them bullies someone else (Figure 1: b → c). In this configuration, it is expected that the second person’s relationship with the third person becomes negative over time (referring to that *i* likes *j* and *i* bullies *h*, then *j* bullies *h*). In other words, when a friend bullies someone, the person will bully that same person as well. This effect may be seen as an influence effect (Rambaran et al., 2015), as it indicates that bullies influence their friends into “agreeing” on whom to victimize. Hence, we call this the *bully influence* hypothesis. It may be explained by the popularity of the bullies. Bullies are generally not well‐liked by peers but can be popular when bullying associates with status in the group (Salmivalli, 2010). For some children, popular peers form role models. They may conform to or mimic their behavior in pursuit of similar status among peers (referring to a social reward; see Brechwald & Prinstein, 2011) or to receive affection from popular friends. Popular kids also possess social strategies and social skills that can be used to persuade or manipulate peers (Sandstrom, 2011).

### Victim Selection and Influence Hypotheses

**Figure 2:**
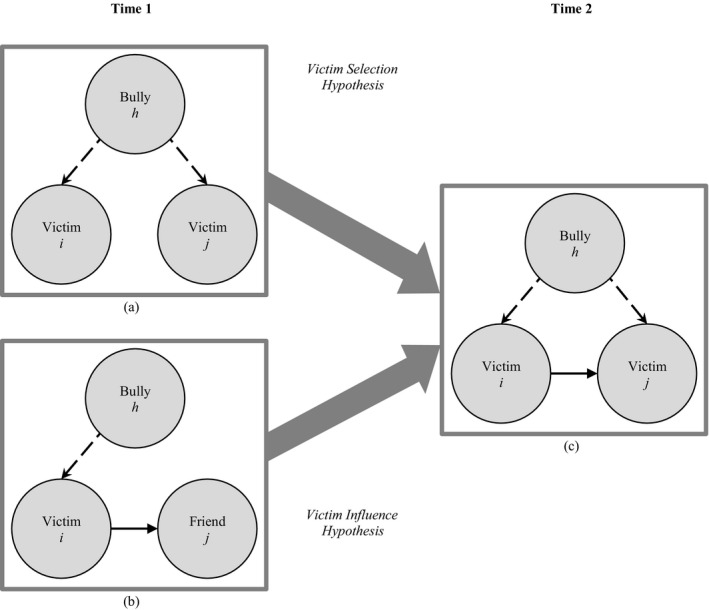
Illustration of the interplay between bullying ("Who do you bully?") and friendship (“Who is your friend?). As can be seen, the same outcome (c) can be produced by different underlying processes. By (a) two children who are victims of the same bully at Time 1 become friends at Time 2 (describing a selection process), and (b) a person who is friends with a victim at Time 1 becomes a victim of the same bully at Time 2 (describing an influence process). To facilitate the interpretation of these network configurations, friendships are represented with solid lines and bullying relationships are represented with dashed lines. This figure is derived from Huitsing et al. (2014).

The third configuration describes a situation where two individuals share the same bully, referring to that they are victimized by the same person (Figure 2: a → c). In this configuration, it is expected that the first person’s relationship with the second person becomes positive over time (referring to that *i* is bullied by *h* and *j* is bullied by *h*, then *i* likes *j*). This selection effect may be explained by bonding over shared negative experiences (a process of co‐rumination; see Rose, 2002). Because rejected children are unattractive and avoided as friends, victims may settle for friendships with each other (a process of default selection; Deptula & Cohen, 2004; Sijtsema, Lindenberg, & Veenstra, 2010). Forming a friendship may also strengthen their position against a common adversary. We call this the *victim selection* hypothesis.

The fourth configuration describes a situation where two individuals are friends, and one of them is victimized by someone else (Figure 2: b → c). In this configuration, it is expected that the second person’s relationship with the third person becomes negative over time (referring to that *i* likes *j* and *i* is bullied by *h*, then *j* is bullied by *h*). In other words, when a friend is bullied by someone, the person will be bullied by that same person as well. A friendship with a victim may weaken one’s own position in the group, thus putting oneself at risk to become the next target. This effect may be seen as an influence effect, as it indicates that there is a contagion of victimization. Accordingly, we call this the *victim influence* hypothesis.

## The Present Study

We investigate bullying relationships together with friendships and use a so‐called multiplex social network approach. We expected that bullies start to become friends over time (H1) and that bullies influence their friends to start bullying the same victim (H2). In addition, we expected that victims start to become friends over time (H3) and that bullies start to victimize the friends of their victims (H4). We tested our hypotheses using longitudinal bivariate social network analysis using SIENA (Simulation Investigation for Empirical Network Analysis; Snijders, Lomi, & Torlo, 2013). This approach allows us to investigate bullying as a network relationship based on nominations for bullying (“By whom are you victimized?”).

## Method

### Sample

Classrooms were drawn from the first three waves of the KiVa study collected in May 2012, October 2012, and May 2013. KiVa is a program aimed to reduce school bullying among children in elementary education (8–12 years) in the Netherlands (Huitsing et al., 2019; Kaufman, Kretschmer, Huitsing, & Veenstra, 2018). A total of 99 schools participated in the study. For this study, we selected the four schools in the control condition that did not combine any classes or grades over the school year (after the summer break in 2012). This ensured us to examine group processes in bullying and victimization in stable classrooms. A description of the program, the control sample, and the intervention sample is described elsewhere (Huitsing et al., 2019; Kaufman et al., 2018; Rambaran, van Duijn, Dijkstra, & Veenstra, 2019a).

We applied an additional set of selection criteria for longitudinal social network analysis to the four selected schools that contained 24 classrooms. First, classroom size had to be larger than 15. Second, classrooms had to contain some stable ties. Third, missingness in network information had to be lower than 20%. This resulted in dropping five classrooms (one classroom had 12 students, two classrooms did not have any stable bullying tie, and two classrooms did not participate at Wave 3). The final sample consisted of 19 classrooms (all single‐grades) with 481 students, of which, at the first wave, consisted of five Grade 2 classrooms (*n* = 96), six Grade 3 classrooms (*n* = 184), three Grade 4 classrooms (*n* = 72), and five Grade 5 classrooms (*n* = 129). The average classroom size was 25 (minimum = 20; maximum = 32). Changes in student classroom composition were minimal (we refer the interested reader to Table S1 for information about each individual classroom). The sample could be characterized as a multi‐age (8–12 year olds) sample of children in elementary school in both urban and rural parts of the Netherlands, with an equal distribution of boys and girls (50.5% boys), composed of 81.8% Dutch, 6.6% Surinamese, 1.3% Moroccan, and 3.1% Turkish, with 5.5% of the children belonging to another Western and 1.7% to another non‐Western ethnic group based on the country of birth of the parents.

### Procedure

Students filled in an Internet‐based questionnaire in their classroom during regular school hours. The process was administered by the teachers, who were present to answer questions and to assist the students when necessary. Prior to the data collection, teachers were given detailed instructions concerning the procedure. During the data collection, support was available through phone and e‐mail.

At the beginning of the questionnaire, students received information about the goal of the study and how to fill in the questionnaire. They were told not to talk to each other or to discuss their answers when they filled out the questionnaire or afterward to ensure each other’s privacy. It was explained to students that their answers would remain confidential. The teachers ensured that students who could not complete the questionnaire on the day of the data collection participated at another day within a month.

Prior to the first measurement (and for students who were new in school, after the first measurement), schools sent information letters to students’ parents. A passive consent procedure allowed students or parents to opt out of student participation. At the start of data collection (2012), universities in the Netherlands did not require institutional review board permission for this type of research. All procedures performed in our study were in accordance with the 1964 Helsinki declaration and its later amendments or comparable ethical standards. A few students did not want to participate, and also a few parents objected to their child’s participation. Overall, the participation rate was high (98.5% at Wave 1).

In an instructional movie, a professional actress explained to students what bullying means, using the following text:

Bullying is when some children repeatedly harass another child. The child who gets bullied has problems defending itself against this. Bullying is not the same as having a fight between two people who are equally strong. Bullying should also not be confused with joking around. Bullying is treating someone repeatedly in a mean way.

Several examples of bullying were given to students, including physical and material forms (e.g., hitting someone, kicking or pinching; stealing or damaging someone’s belongings) and relational and verbal forms (e.g., making fun of someone, calling names, saying mean things; gossip about someone; excluding from social activities).

### Measures

#### Bullying Networks

Bullying was measured with network nominations for peer bullying. At each time point, students were asked to indicate if they were victimized by classmates, and if so, were presented with a roster showing the names of all classmates with the accompanying text: “The following two questions are about who starts the bullying. Often, certain classmates initiate the bullying and others join them.” For bullying initiation, they were asked: “Who starts bullying you?” For bullying assisting, they were asked: “Who always joins the bully or is there when the bullying takes place?”

The two items were collapsed into one measure. The overlap between nominations for initiation and assisting was on average 22.7% over the waves, indicating that there is some overlap at the dyadic level between initiating and assisting in bullying. Nominations for the one measure of bullying were reversed so that when *i* considers *j* as his or her bully, this translates into *j* bullying *i* (from the victim’s perspective). The perception and experience of victims are important in bullying research. For that reason, we look at bullying from the point of view of the victim. Bullying nominations were measured as present (1) or absent (0). Students who indicated not being victimized by classmates did not fill out the nomination questions. Their “answers” were considered as absent nominations. Based on these nominations, bullying networks were obtained containing all directed bully nominations for each classroom.

#### Friendship Networks

Friendship was measured with network nominations. In all waves, students were presented with a roster showing the names of all classmates and asked whom of their classmates they considered as their best friends (“Who are your best friends?”). Students could choose as many same‐sex and other‐sex classmates as they wished. Friendship nominations were coded 1 and non‐nominations were coded 0, resulting in friendship networks consisting of directed nominations for each classroom.

### Analytic Strategy

Analyses were conducted using longitudinal bivariate social network analysis (see for an introduction to this model: Snijders et al., 2013), which allowed for examination of the development of children’s friendship and bullying networks simultaneously over time and their interplay (Snijders et al., 2013) while taking structural and individual effects into account. This method has been used before to examine dependencies between positive and negative type networks in (school) classrooms, most prominently friendship and dislike (e.g., Berger & Dijkstra, 2013; Pál, Stadtfeld, Grow, & Takács, 2016; Rambaran et al., 2015; with the exception of Huitsing et al., 2014 who examined dependencies between defending and victimization).

The effects were first analyzed for each classroom model separately, combined over the two observation periods (Time 1 and 2; Time 2 and 3), and were then meta‐analyzed in R (Viechtbauer, 2010). No clear pattern with regard to differences in selection and influence effects were found across time (see Table S4). Each classroom model was estimated with the same specification (see Table S2). Parameters were fixed in classrooms where the accompanying statistics (network) configurations were absent. All models showed good convergence (Ripley, Snijders, Boda, Vörös, & Preciado, 2019). In some classroom networks, one or two additional effects were necessary to achieve an acceptable model fit, which is standard practice in social network analysis for bullying and victimization networks (Huitsing et al., 2014; Rambaran, van Duijn, Dijkstra, & Veenstra, 2019b; Ripley et al., 2019). Initially, a good fit was obtained for 12 of 19 classrooms, then two more classrooms obtained good fit by including additional effects (classrooms 11 and 15). The remaining five classrooms (3, 12, 13, 14, and 17) had no optimal fit (why these particular classrooms had no optimal fit can be seen in Table S3 and Figure S1; the notes of Table S3 provide a detailed explanation). Sensitivity analysis leaving out these five classrooms yielded substantially the same results. These classrooms were therefore not excluded from the meta‐analysis (compare Table S5 with Table 2).

### Model Specification and Effect Interpretation

The bivariate analysis in RSiena yields three types of parameters: rate parameters for each type of network (referring to friendship and bullying), selection parameters for each type of network, and between‐networks parameters, reflecting the interplay between the two networks. This model specification was largely derived from Huitsing et al. (2014) and Rambaran et al. (2015). The choice of these parameters was based on a combination of three requirements: (a) to include structures that are theoretically relevant for hypotheses testing, (b) to capture adequately the structures in our networks, and (c) to keep the models parsimonious and similar across the classrooms. The final two requirements were assessed using fit statistics and model convergence.

For the structural part of the model for friendship dynamics and bullying dynamics, we included the basic effects (outdegree and reciprocity, indegree popularity, and outdegree activity), network closure effects (actors at Distance 2 and transitive triplets), and subgroup effects (four cycles). For the structural part of the model for bullying dynamics, we also included effects that inversely represent network isolates (indegrees and outdegrees of at least 1). A dyadic covariate effect was included to control for same‐sex friendship and bullying.

For between‐networks effects, the network in the role of dependent variable is denoted by X and the network in the role of explanatory variable by W (see Table S2). We tested the dependence of one type of network on another type, controlling for dyadic and degree‐related multiplex effects to account for main effects of one network type on another and dynamics in in‐ and outdegrees between network types and their covariance. The *shared victim to friendship* effect indicates that “agreeing” upon shared victims will result in the formation of a new friendship tie (Figure 1: a → c) to test the bully selection hypothesis (H1). The *friendship agreement to bullying* effect indicates that an existing friendship tie will result in the formation of a new bullying tie (Figure 1: b → c) and tested the bully influence hypothesis (H2). The same configurations in reversed direction tested the victim selection hypothesis (H3: *shared bully to friendship* effect; Figure 2: a → c) and the victim influence hypothesis (H4: *friendship agreement to victimization* effect; Figure 2: b → c).

## Results

### Descriptive Findings

Table 1 presents the summarized descriptive findings for the 19 classrooms. Table S1 reports information per classroom. On average, participants indicated to have five friends and reported to have two bullies (note that this number is higher when counted among the victims; see average degrees in Table 1). The majority of students nominated at least one friend (93%–94%) or were reported by their peers to bully at least one classmate (64%–66%); over one‐third were victims (37%–47%). Density (proportion of nominations given) was higher for friendship (0.21–0.22) than for bullying (0.08–0.09), indicating that bullying networks were sparser. The percentage of reciprocated nominations was also higher for friendships (34%–72%) than for bullying (0%–43%). Over 75% of friendship nominations and 50% of bullying nominations were same sex, and boys bullied girls more often than vice versa. Indirect ties and transitivity were more common for friendships than for bullying (see Table 1).

**Table 1:** Descriptive Statistics of the Friendship Networks and Bullying Networks Per and Between Time Points (19 Classrooms, 481 Students)

	Friendship networks			Bullying networks		
	Wave 1	Wave 2	Wave 3	Wave 1	Wave 2	Wave 3
Density indicators						
Density^a^	0.22 (0.15 to 0.33)	0.22 (0.13 to 0.31)	0.21 (0.12 to 0.30)	0.09 (0.02 to 0.22)	0.08 (0.01 to 0.17)	0.08 (0.01 to 0.18)
Number of ties	135 (108 to 206)	136 (82 to 226)	127 (70 to 185)	52 (14 to 115)	49 (6 to 100)	49 (10 to 93)
At least one out‐tie	0.94 (0.80 to 1)	0.93 (0.73 to 1)	0.94 (0.69 to 1)	0.65 (0.37 to 0.95)	0.64 (0.19 to 0.85)	0.66 (0.26 to 0.95)
At least one in‐tie	0.95 (0.84 to 1)	0.94 (0.81 to 1)	0.93 (0.73 to 1)	0.47 (0.26 to 0.84)	0.40 (0.15 to 0.60)	0.37 (0.17 to 0.58)
Average degree^b^	5.3 (4.1 to 7.8)	5.3 (3.2 to 7.4)	5.0 (3.0 to 7.2)	2.1 (0.5 to 4.6)	2.0 (0.2 to 4.0)	2.0 (0.4 to 4.0)
*SD* outdegree	3.9 (2.2 to 6.5)	3.9 (2.7 to 5.6)	3.4 (1.9 to 6.1)	2.1 (0.8 to 4.4)	2.0 (0.5 to 3.4)	2.0 (0.8 to 3.6)
*SD* indegree	2.9 (2.0 to 4.0)	2.8 (1.5 to 3.9)	2.7 (1.6 to 3.9)	2.9 (1.2 to 4.6)	3.0 (0.7 to 5.5)	3.4 (0.9 to 5.5)
Dyadic indicators						
Asymmetrical ties	122 (80 to 272)	130 (60 to 252)	116 (56 to 198)	88 (24 to 178)	85 (12 to 158)	85 (20 to 126)
Mutual ties	74 (44 to 122)	71 (46 to 100)	69 (36 to 102)	8 (0 to 32)	6 (0 to 26)	7 (0 to 40)
At least one mutual tie	0.94 (0.80 to 1)	0.93 (0.73 to 1)	0.94 (0.69 to 1)	0.65 (0.37 to 0.95)	0.64 (0.19 to 0.85)	0.66 (0.26 to 0.95)
Reciprocity^c^	0.55 (0.34 to 0.71)	0.53 (0.41 to 0.63)	0.55 (0.39 to 0.72)	0.13 (0.00 to 0.33)	0.12 (0.00 to 0.26)	0.10 (0.00 to 0.43)
Sex combinations						
Boy–boy	0.35 (0.19 to 0.55)	0.39 (0.22 to 0.63)	0.37 (0.22 to 0.56)	0.33 (0.00 to 0.85)	0.34 (0.04 to 0.76)	0.32 (0.00 to 0.78)
Girl–girl	0.44 (0.23 to 0.63)	0.41 (0.13 to 0.59)	0.44 (0.18 to 0.65)	0.20 (0.05 to 0.36)	0.19 (0.00 to 0.50)	0.15 (0.00 to 0.38)
Boy–girl	0.10 (0.04 to 0.21)	0.10 (0.00 to 0.25)	0.09 (0.02 to 0.27)	0.34 (0.03 to 0.57)	0.37 (0.08 to 0.68)	0.39 (0.08 to 0.79)
Girl–boy	0.11 (0.02 to 0.31)	0.09 (0.00 to 0.23)	0.10 (0.00 to 0.22)	0.14 (0.00 to 0.44)	0.10 (0.00 to 0.35)	0.14 (0.00 to 0.32)
Triadic indicators						
Distance 2 (indirect ties)^d^	0.92 (0.72 to 1)	0.91 (0.62 to 1)	0.90 (0.58 to 1)	0.52 (0.16 to 0.95)	0.49 (0.04 to 0.85)	0.55 (0.00 to 0.95)
Transitivity index^e^	0.51 (0.37 to 0.70)	0.55 (0.39 to 0.74)	0.51 (0.38 to 0.71)	0.38 (0.00 to 0.79)	0.45 (0.00 to 0.75)	0.49 (0.14 to 1)

	Wave 1 to 2	Wave 2 to 3	Wave 1 to 2	Wave 2 to 3
Change indicators				
Creating ties (0 → 1)	55 (20 to 115)	46 (15 to 90)	32 (2 to 86)	29 (6 to 60)
Dissolving ties (1 → 0)	54 (34 to 112)	55 (31 to 108)	35 (10 to 90)	29 (5 to 81)
Stable ties (1 → 1)	81 (60 to 139)	81 (46 to 124)	17 (1 to 41)	20 (1 to 43)
Jaccard index^f^	0.43 (0.32 to 0.52)	0.45 (0.29 to 0.56)	0.21 (0.02 to 0.43)	0.24 (0.03 to 0.54)

	Friendship × Bullying			Friendship × Victimization		
	Wave 1	Wave 2	Wave 3	Wave 1	Wave 2	Wave 3
Dependence indicators						
Moran’s *I* autocorrelation^g^	0.18 (−0.12 to 0.56)	0.20 (−0.01 to 0.59)	0.22 (0.04 to 0.58)	−0.01 (−0.11 to 0.14)	−0.01 (−0.19 to 0.21)	−0.03 (−0.15 to 0.14)
Jaccard index^h^	0.06 (0.02 to 0.14)	0.06 (0.01 to 0.09)	0.06 (0.01 to 0.12)	—	—	—
Shared bullies/victims^i^	0.37 (0.23 to 0.67)	0.42 (0.26 to 1)	0.35 (0.17 to 0.57)	0.23 (0.00 to 0.48)	0.22 (0.00 to 0.47)	0.17 (0.04 to 0.36)

Note

The table shows averages. Minimum and maximum are shown in parentheses.

a Density is the number of observed ties divided by the total number of possible ties.

b If counted among the victims (referring to students who nominated at least one bully in class), average degree is 4.4 (min = 1.9, max = 8.4) at W1, 4.8 (min = 1.5, max = 8.5) at W2, and 5.2 (min = 2, max = 7.8) at W3, indicating that victims had, on average about 4–5 bullies in class.

c Reciprocity was calculated as 2*M*/(2*M* + *A*), where *M* = mutual ties and *A* = asymmetric ties.

d Distance 2 is the proportion of respondents with ties at two degrees of separation (with at least one connecting intermediary).

e Transitivity was calculated as the number of transitive triplets divided by the number of two‐paths (or two‐stars).

f Jaccard index is the proportion of stable ties in relation to creating ties and dissolving ties.

g Moran’s *I *autocorrelation indicates the level of similarity (as correlation) between friendship and bullying (outdegrees) or victimization (indegrees).

h Jaccard index is the degree (proportion) of dyadic overlap between friendship networks and bullying networks.

i Proportion of shared outgoing W‐ties (*i* and *j* bully the same victim *h*) and incoming W‐ties (*i* and *j* are victimized by the same bully *h*) for which there are also outgoing X‐ties (*i* and *j* are friends).

Part 2 of Table 1 shows information about tie stability in friendship and bullying. As shown by the Jaccard indices, all classrooms had sufficient stability in friendship ties between time points (29%–56%), but stability in bullying ties was at the most 43% and almost absent in some classrooms. This means that students changed bullying ties more frequently than friendship ties from one time point to the next. For some classrooms, high turnover rates in combination with the model’s complexity led to convergence issues. In these instances, we followed recommendations in the RSiena manual (Ripley et al., 2019) and fixed the number of opportunities to change bullying ties at the observed value (see Table S2).

Dependencies between friendship and bullying or victimization were assessed in three ways (see Part 3 in Table 1). Moran’s *I* network autocorrelation coefficient indicates the degree to which friends are similar in bullying or victimization (Steglich, Snijders, & Pearson, 2010). Values close to 0 are expected under random pairing (referring to perfect independence), whereas values close to 1 indicate perfect similarity. Typically, values of 0.2 to 0.3 indicate clear behavioral similarity. In our study, for most classrooms, the degree of similarity in bullying between friends was modest as indicated by positive, moderate values of Moran’s *I* network autocorrelation coefficients (Steglich et al., 2010), whereas similarity in victimization was weak or absent. The amount of overlap between friendship and bullying was generally low, meaning that friends did not bully each other. Finally, friendships occurred more among bullies with the same victims than among victims with the same bullies.

### SIENA Findings

Table 2 presents the summary of the multivariate RSiena analyses (performed with RSiena version 1.2‐4). Figure S2 shows the results per classroom. The first column in Table 2 shows the mean estimates with friendships as the outcome, whereas the second column shows the mean estimates with bullying as the outcome. The results comprise two main parts: effects describing structural network dynamics for friendship and bullying separately; effects describing dynamics in dependencies between friendship and bullying.

**Table 2:** Results From Longitudinal Multivariate Network Models Predicting Co‐Evolution of Friendship and Bullying (19 Classrooms, 481 Students)

	Hypothetical change			Friendship networks			Bullying networks		
	t*x*	→	t*x* + *m*	Est.	*SE*	*n*	Est.	*SE*	*n*
Effect parameters									
Rate effects									
Network rate t1 → t2				8.01^f^, ***	0.52^a^, ^a^	19	7.29^f^, ***	1.12^a^, ^a^	14
Network rate t2 → t3				8.08^f^, ***	0.57^a^, ^a^	19	6.82^f^, ***	0.77^a^, ^a^	13
Structure effects									
Outdegree (density)	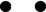	→	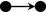	−2.65^f^, ***	0.21	19	−2.17^f^, ***	0.30^a^, ^a^	19
Outdegree isolates	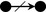	→	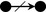				−1.58^f^, ***	0.31^a^, ^a^	18
Indegree isolates	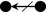	→	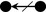				−2.41^f^, ***	0.25	18
Reciprocity	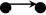	→	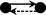	1.61^f^, ***	0.14	19	0.37+	0.19	18
Indegree popularity	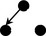	→	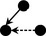	0.07	0.06	18	0.02	0.07	19
Outdegree activity	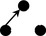	→	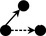	0.02	0.04	17	0.10	0.08	17
Transitive triplets	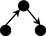	→	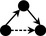	0.27^f^, ***	0.08	19	0.04	0.13	19
Transitive reciprocated triplets	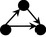	→	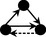	−0.34^f^, ***	0.09	19			
Actors at distance 2	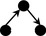	→	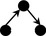	−0.20^e^, **	0.07	19	−0.24+	0.14	18
Four‐cycles	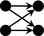	→	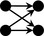	−0.02	0.04	17	−0.07	0.07	17
Sex effects									
Same‐sex	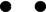	→	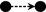	0.63^f^, ***	0.12	19	0.12	0.14	19
Dyadic multiplex effects									
Existing tie W → new tie X	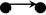	→	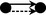	−0.20	0.31	18	−0.04	0.24	18
Degree‐related multiplex effects									
Indegree tie W → Indegree tie X	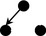	→	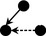	−0.002	0.06	18	−0.05	0.08	18
Outdegree tie W → Indegree tie X	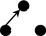	→	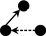	−0.15+	0.09	19	−0.02	0.09	19
Outdegree tie W → Outdegree tie X	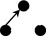	→	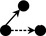	−0.05	0.08	18	−0.15+	0.08	19
Mixed triadic multiplex effects^b^, ^h^, ^b^									
H1: shared victim to friendship	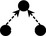	→	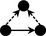	0.41^d^, *	0.17	17			
H2: friendship agreement to bullying	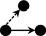	→	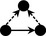				0.74^f^, ***	0.14	19
H3: shared bully to friendship	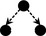	→	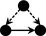	0.06	0.10	17			
H4: friendship agreement to victimization	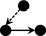	→	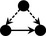				0.03	0.12	18

Note.

Significance tests performed by dividing the estimates with its standard error resulting in *t*‐values which under the null hypothesis are approximately normally distributed (Ripley et al., 2019). Convergence statistics: *t* ratios all < .07; overall maximum convergence ratio < .21.

a Significant differences between classrooms.

^b^ To facilitate the interpretation of these network configurations, friendships are represented with solid lines and bullying relationships are represented with dashed lines.

^+^ *p *≤ .10.

* *p* ≤ .05.

** *p* ≤ .01.

*** *p* ≤ .001 (two‐tailed test).

#### Structural Network Effects

Participants were likely to be selective in their nominations (negative outdegree effects): They did not become friends with or bullied everyone (as indicated by the victims). Some students were uninvolved in bullying relationships: They were neither victimized (negative indegree isolate effect) nor bullied others (negative outdegree isolate effect). Nominations received were likely to be reciprocated (positive reciprocity effects). Furthermore, participants tended to nominate friends of friends as friends (positive transitive triplets effect) and to avoid having indirect connections (negative Distance 2 effects). Friendships were often same sex, whereas bullying was directed to same‐ as well as cross‐sex peers.

#### Between‐Networks Effects: Bully Selection and Influence Hypotheses

These were tested by two between‐networks effects (see Table 2; see also Figure 1). Consistent with the bully selection hypothesis, the *shared victim to friendship* effects were positive in all classrooms (with the exception of one classroom; see Figure S2.P with friendship as outcome): When two children bullied the same victim they were likely to become friends (see Table 2: Est. = .41, *p* < .05). In line with the bully influence hypothesis, the *friendship agreement to bullying* effects were positive in all classrooms (see Figure S2.Q with bullying as the outcome): Children were likely to start bullying the classmates who were bullied by their friends (see Table 2: Est. = .74, *p* < .001).

#### Victim Selection and Influence Hypotheses

These were tested by the matching between‐networks effects in the opposite direction (see Table 2; see also Figure 2). The victim selection hypothesis was not supported: The mean estimate of the *shared bully to friendship* effect was close to zero and nonsignificant (see Table 2: Est. = .06, *p* = .53). There was on average no indication that two children victimized by the same bully were likely to become friends (see Figure S2.Q with friendship as outcome). In addition, the victim influence hypothesis was not supported: The mean estimate of the *friendship agreement to victimization* effect was close to zero and nonsignificant (see Table 2: Est. = .03, *p* = .81). There was no indication that children were likely to become victimized by the bullies of their friends (see Figure S2.R with bullying as the outcome).

## Discussion

We examined the interplay between friendship and bullying ties in a sample of children in 19 elementary school classrooms over 1 year. Based on the idea that bullying is a group process, it was expected that friendships would be formed when two children bullied the same person (bully selection hypothesis) and that children would start to bully the victims of their friends (bully influence hypothesis). Similarly, it was expected that friendships would be formed when two children were victimized by the same bully (victim selection hypothesis) and that children would become victimized by the bullies of their friends (victim influence hypothesis). Longitudinal bivariate social network analysis provided evidence for the first two hypotheses but not for the latter two.

### Bully Selection and Influence

The finding that two children are likely to become friends when they bully the same classmate suggests that sharing a “common target” promotes friendship. From a developmental perspective, friendships serve different functions and have different meanings for children across different stages of child development (Rubin, Bowker, McDonald, & Menzer, 2013; Rubin, Bukowski, & Parker, 2006), and based on this there may be several plausible explanations for this finding. In middle childhood, children describe their friends as those who are enjoyable and rewarding to be with. Like most other children, bullies also want social affection from their peers (Veenstra et al., 2010), and being in a friendship with someone that bullies the same victim might serve that specific purpose as these bullies are likely to be closer to each other and more in contact. Bullies may also have shared values about their own and other’s bullying behavior, bullying attitudes, and morality, and this is central to children’s conceptions of friendship, particularly because bullying behavior is considered as non‐normative behavior. Accordingly, bullies might reward loyal peers and help them deal with others, for instance, by sticking up for them or becoming friends. A friendship might thus also protect from others who stand up to them by defending the victim and secure a strong position in the group (Huitsing et al., 2014; Sainio et al., 2011), which may discourage others to side with the victim because of fear to become victims themselves (Pozzoli & Gini, 2010). Some children might also want to become friends with the bullies, particularly because bullies are often considered as popular among peers (Salmivalli, 2010; Veenstra et al., 2010). It is possible that these children try to become similar to the bullies are (in their attitudes, likes, and dislikes). One way to demonstrate similarity and to get the bullies’ acceptance is to bully together with them. As a result, the friendship is formed, because the bullies like “joiners.”

Our finding that children are likely to start to bully the same victims as their friends indicates that children “agree” with their friends on whom to target. This may be explained by bullies influencing their friends on whom to victimize. Bullying is often associated with high social status (Salmivalli, 2010; Veenstra et al., 2010), and for that reason, children who are friends with bullies may conform to their behavior by joining in the bullying. This finding adds to our current understanding of bullying as a group process, by showing that friends play an important role in the bullying process. This is in line with previous research that shows that peers play a critical role in the development of bullying over and beyond child and family factors (Pepler, Jiang, Craig, & Connolly, 2008). It may provide behavioral confirmation, affection, and social status to the bullies, thereby forming a strong motivation to continue bullying (Salmivalli, 2010; Veenstra et al., 2010).

### Victim Selection and Influence

Unlike previous network studies that found friendship selection and influence in victimization in adolescence (Lodder, Scholte, Cillessen, & Giletta, 2014; Sentse, Dijkstra, Salmivalli, & Cillessen, 2013; Sijtsema, Rambaran, & Ojanen, 2013), our results did not provide evidence for the same processes in childhood. This may be explained by age or developmental differences. In our childhood sample, victimization was common as many children indicated being bullied by peers. In early adolescence, the number of bullies increases or remains stable, whereas the number of victims decreases (Nansel et al., 2001; Pellegrini & Long, 2002). This increase in bullying during early adolescence is followed by a decrease during mid‐ and late adolescence (Kretschmer, Veenstra, Deković, & Oldehinkel, 2017). This suggests that victims are especially in a weak position in early adolescence when bullying peaks. It may also be that victims in childhood are not strategic enough to befriend co‐victims, which may be a way to create social support and stop the bullying. Finally, children in elementary school have a history together. They know each other quite often since kindergarten. They often live in the same neighborhood where victims may still be connected to nonvictims. However, in secondary education, the nonvictims may no longer want to be associated with victims, as the importance of status increases. We note, however, that we only included four schools. Therefore, this explanation may not be generalizable, and this should be taken into account. For instance, in urban areas or large cities in the USA students may undergo multiple school transitions, from kindergarten to elementary school to middle or junior high school. In these situations, it is likely that children have less of a shared history together during elementary education.

### Change in Bullying Networks

We noticed that the change in bullying ties is high (see also Huitsing et al., 2014; Rambaran et al., 2019b). Recent work on the dynamic interplay between bullying and popularity showed that bullies frequently change victims to maintain a high social status (van der Ploeg, Steglich, & Veenstra, 2019). Apparently, repeatedly bullying the same victim loses its “effect” after some time, and bullies seek out new victims. This also underlines that bullies are goal oriented and strategic (Salmivalli, 2010; Veenstra et al., 2010). It further points out that future research should incorporate the role of status or popularity as attributes of children when examining bullying in friendship networks.

### Sex Segregation Friendship and Bullying Networks

Friendship networks were largely sex segregated, whereas bullying was as often directed to same‐ as to cross‐sex peers. Considerable work has demonstrated that children develop largely in segregated social worlds (Maccoby, 1998). Our findings suggest that excessive bullying relationships between the two sexes may be a reason for this: Girls may not want to befriend boys because they may also be bullied by them, driving girls further apart from the boys in terms of friendship. This is in line with studies showing that boys and girls accept same‐sex classmates over cross‐sex classmates, that boys are more often nominated than girls as bully perpetrators against both sexes, and that as a consequence boys who bully are rejected by girls for whom they pose a potential threat (Veenstra et al., 2010; Veenstra, Verlinden, Huitsing, Verhulst, & Tiemeier, 2013). Boys who bully may target girls, to maximize their gains in social status, while minimizing loss of affection or rejection from other bullying boys (Sainio, Veenstra, Huitsing, & Salmivalli, 2012). Compared to boys, girls who bully do so more relationally (e.g., social exclusion, gossiping; Espelage, Mebane, & Swearer, 2004), which makes it also less noticeable for other bullying girls who want to seek out their companion or friendship. An avenue for future research would be to take into account potential gendered processes in both friendship and bullying networks to better understand how bullying relationships unfold in friendship networks.

### Limitations and Directions for Future Research

Selecting stable classrooms greatly facilitated our examination of (peer) group processes in bullying and victimization, but many classrooms were left out from schools that combined one or more classes or grades. Most school children across the world are traditionally organized in same‐age/grade classrooms (Mulryan‐Kyne, 2007; Veenman, 1995), and our findings may only be applicable to these specific type of classrooms. However, there are also children who interact within mixed‐age/grade peer groups as some schools combine different grades within one group, so‐called multigrade or multiage classrooms (Mulryan‐Kyne, 2007; Veenman, 1995). Multigrade classrooms are prominent in the Netherlands. In our sample, only four schools in the control condition did not combine any classes or grades over the school year. These classrooms may exhibit different group dynamics (Ellis et al., 2012). For instance, bullying occurs more frequently within the same grade (Rambaran et al., 2019a). Bullying is also not limited to classroom boundaries: They occur outside of classroom too (Huitsing et al., 2014). Hence, school‐level or grade‐level analyses would capture a larger proportion of children’s bullying networks (Rambaran et al., 2019b). Victims in similar positions may find friends in the wider school network.

Although our study included a broader definition of bullying relationships by including both bullying initiation and bullying assisting, we did not include bystanders in our analysis. Following the participant roles approach (Salmivalli et al., 1996), the behavior of bystanders in the classroom matters for the occurrence and continuation of bullying. For instance, with many defenders in the classroom, victims receive more support from peers (defending the victim), and the frequency of bullying is lower (Salmivalli, Voeten, & Poskiparta, 2011). In contrast, when there are many reinforcers of the bullies, the frequency of bullying is higher. This suggests that bullying relationships are affected by the classroom context in terms of group norms (Sentse, Scholte, Salmivalli, & Voeten, 2007). Moreover, children’s positions in the classroom are also affected by their social status among peers, in terms of acceptance and rejection (Veenstra et al., 2010). A more comprehensive study of the group process of bullying would take into account the role of bystanders, social status, and group norms.

By incorporating a relational perspective to bullying, *who bullies whom*, we were able to test more subtle or intricate hypotheses concerning selection and influence processes resulting from the interplay between bullying relationships and friendship relationships. Even though we were able to link the bullying behavior of children to that of their friends, we do not know exactly *why* children (started to) bully their classmates, however. Similarly, we do not know precisely why bullies with the same victims befriended each other or why victims with the same bullies did not befriend each other. More detailed information about the specific reasons resulting in friendship selection and influence would allow for a better understanding of the mechanisms behind the group process of bullying.

### Conclusion

In sum, our study provides evidence for group processes in bullying networks in childhood. On the one hand, bullies are likely to select each other as friends; on the other hand, bullies are likely to influence their friends to start bullying. These novel findings highlight how group processes in bullying operate through children’s peer networks in the classroom.

## Supporting information


**Figure S1** **.** Visualization of Goodness of Fit Statistics for Models Summarized in Table S3
**Figure S2** **.** Distribution of Parameter Estimates and Standard Errors for Models Summarized in Table 2
**Table S1** **.** Overview of Individual and Classroom Information for Descriptive Statistics Summarized in Table 1
**Table S2** **.** Effects Included for Models Summarized in Table 2
**Table S3** **.** Goodness of Fit Statistics for Models Summarized in Table 2
**Table S4** **.** Time Heterogeneity Tests of Selection Effects and Influence Effects
**Table S5** **.** Sensitivity Analysis Excluding the Five Classrooms With No Optimal Fit
