# Supplementary material for: Bullying as a Group Process in Childhood: A Longitudinal Social Network Analysis
Source: Child Dev. 2019 Aug 19;91(4):1336–52. doi: 10.1111/cdev.13298 (PMC7496633; doi:10.1111/cdev.13298)
Supplement: Supplementary file 1 — Figure S1 . Visualization of Goodness of Fit Statistics for Models Summarized in Table S3 Figure S2 . Distribution of Parameter Estimates and Standard Errors for Models Summarized in Table 2 Table S1 . Overview of Individual and Classroom Information for Descriptive Statistics Summarized in Table 1 Table S2 . Effects Included for Models Summarized in Table 2 Table S3 . Goodness of Fit Statistics for Models Summarized in Table 2 Table S4 . Time Heterogeneity Tests of Selection Effects and Influence Effects Table S5 . Sensitivity Analysis Excluding the Five Classrooms With No Optimal Fit [file CDEV-91-1336-s001.docx]

Supporting Information to:

**‘Bullying as a Group Process in Childhood: A Longitudinal Social Network Analysis’**

Authored by:

Ashwin Rambaran, Jan Kornelis Dijkstra, René Veenstra

List of Tables and Figures:

**Table S1** Overview of individual and classroom information for descriptive statistics summarized in Table 1.

**Table S2** Effects included for models summarized in Table 2.

**Table S3** Goodness of Fit statistics for models summarized in Table 2.

**Figure S1** Visualization of Goodness of Fit statistics for models summarized in Table S3.

**Figure S2** Distribution of parameter estimates and standard errors for models summarized in Table 2.

**Table S4** Time heterogeneity tests of selection effects and influence effects.

**Table S5** Sensitivity analysis excluding the five classrooms with no optimal fit.

| **Table S1** Overview of individual and classroom information for descriptive statistics summarized in Table 1. | | | | | | | | | | | | | | | | | | | | |
| --- | --- | --- | --- | --- | --- | --- | --- | --- | --- | --- | --- | --- | --- | --- | --- | --- | --- | --- | --- | --- |
| *Classroom* | | 1 | 2 | 3 | 4 | 5 | 6 | 7 | 8 | 9 | 10 | 11 | 12 | 13 | 14 | 15 | 16 | 17 | 18 | 19 |
| Total size^a^ | | 25 | 30 | 20 | 29 | 22 | 24 | 25 | 27 | 24 | 24 | 26 | 26 | 25 | 23 | 23 | 28 | 25 | 23 | 32 |
| Grade^b^ | | 2 (3) | 3 (4) | 4 (5) | 5 (6) | 2 (3) | 2 (3) | 3 (4) | 3 (4) | 3 (4) | 4 (5) | 5 (6) | 5 (6) | 2 (3) | 3 (4) | 3 (4) | 4 (5) | 5 (6) | 5 (6) | 3 (4) |
| Age (in years) | | 8.2 | 9.4 | 10.5 | 11.3 | 8.2 | 8.2 | 9.1 | 9.2 | 9.2 | 10.5 | 11.4 | 11.5 | 8.2 | 9.3 | 9.2 | 10.1 | 11.4 | 11.1 | 9.2 |
| Boys | | 60.0% | 40.0% | 45.0% | 41.4% | 45.5% | 54.2% | 56.0% | 63.0% | 41.7% | 45.8% | 38.5% | 50.0% | 32.0% | 60.9% | 56.5% | 50.0% | 52.0% | 65.2% | 62.5% |
| *Respondents^c^* | |  |  |  |  |  |  |  |  |  |  |  |  |  |  |  |  |  |  |  |
| Present | |  |  |  |  |  |  |  |  |  |  |  |  |  |  |  |  |  |  |  |
|  | W1 | 23 | 30 | 20 | 29 | 22 | 24 | 25 | 26 | 24 | 23 | 25 | 24 | 24 | 23 | 22 | 26 | 23 | 23 | 30 |
|  | W2 | 25 | 30 | 20 | 29 | 21 | 21 | 21 | 27 | 23 | 24 | 26 | 20 | 22 | 23 | 21 | 28 | 25 | 23 | 32 |
|  | W3 | 25 | 30 | 19 | 29 | 21 | 21 | 22 | 26 | 24 | 24 | 26 | 20 | 22 | 22 | 22 | 28 | 25 | 23 | 31 |
| Missing | |  |  |  |  |  |  |  |  |  |  |  |  |  |  |  |  |  |  |  |
|  | W1 | 0 | 0 | 0 | 0 | 0 | 0 | 0 | 0 | 0 | 1 | 1 | 0 | 1 | 0 | 0 | 1 | 0 | 0 | 1 |
|  | W2 | 0 | 0 | 0 | 0 | 0 | 1 | 3 | 0 | 1 | 0 | 0 | 3 | 0 | 0 | 1 | 0 | 0 | 0 | 0 |
|  | W3 | 0 | 0 | 1 | 0 | 0 | 0 | 2 | 1 | 0 | 0 | 0 | 1 | 0 | 0 | 0 | 0 | 0 | 0 | 0 |
| Joining | |  |  |  |  |  |  |  |  |  |  |  |  |  |  |  |  |  |  |  |
|  | W2 | 2 | 0 | 0 | 0 | 0 | 0 | 0 | 1 | 0 | 0 | 0 | 0 | 0 | 0 | 0 | 1 | 2 | 0 | 1 |
|  | W3 | 0 | 0 | 0 | 0 | 0 | 0 | 0 | 0 | 0 | 0 | 0 | 0 | 0 | 0 | 0 | 0 | 0 | 0 | 0 |
| Leaving | |  |  |  |  |  |  |  |  |  |  |  |  |  |  |  |  |  |  |  |
|  | W2 | 0 | 0 | 0 | 0 | 1 | 2 | 1 | 0 | 0 | 0 | 0 | 1 | 3 | 0 | 1 | 0 | 0 | 0 | 0 |
|  | W3 | 0 | 0 | 0 | 0 | 0 | 1 | 0 | 0 | 0 | 0 | 0 | 2 | 0 | 1 | 0 | 0 | 0 | 0 | 1 |
| Staying | |  |  |  |  |  |  |  |  |  |  |  |  |  |  |  |  |  |  |  |
|  | W2 | 23 | 30 | 20 | 29 | 21 | 22 | 24 | 26 | 24 | 24 | 26 | 25 | 22 | 23 | 22 | 27 | 23 | 23 | 31 |
|  | W3 | 25 | 30 | 20 | 29 | 22 | 23 | 25 | 27 | 24 | 24 | 26 | 24 | 25 | 22 | 23 | 28 | 25 | 23 | 31 |
| *Average Degree^d^* | |  |  |  |  |  |  |  |  |  |  |  |  |  |  |  |  |  |  |  |
| Friendship | |  |  |  |  |  |  |  |  |  |  |  |  |  |  |  |  |  |  |  |
|  | W1 | 4.3 | 4.7 | 6.1 | 4.1 | 5.2 | 5.3 | 6.0 | 6.2 | 4.5 | 5.0 | 4.3 | 5.2 | 7.8 | 5.5 | 4.7 | 5.6 | 4.4 | 6.1 | 6.4 |
|  | W2 | 7.4 | 4.9 | 4.6 | 4.3 | 5.2 | 4.6 | 3.7 | 5.9 | 5.5 | 6.3 | 5.7 | 3.2 | 6.0 | 4.8 | 4.1 | 6.7 | 4.9 | 6.6 | 7.1 |
|  | W3 | 7.2 | 4.9 | 3.5 | 3.7 | 4.5 | 4.6 | 3.7 | 5.6 | 3.1 | 5.3 | 5.0 | 3.0 | 5.0 | 5.9 | 3.9 | 6.6 | 6.9 | 6.7 | 5.8 |
| Bullying | |  |  |  |  |  |  |  |  |  |  |  |  |  |  |  |  |  |  |  |
|  | W1 | 4.6 | 1.0 | 4.2 | 3.6 | 3.0 | 0.7 | 1.4 | 2.2 | 1.7 | 4.2 | 0.5 | 1.7 | 1.7 | 1.9 | 1.4 | 2.0 | 1.3 | 1.7 | 1.1 |
|  | W2 | 1.7 | 1.2 | 2.7 | 2.1 | 2.1 | 2.0 | 4.0 | 1.3 | 1.7 | 3.0 | 0.2 | 2.5 | 1.8 | 2.5 | 1.0 | 3.0 | 1.4 | 2.0 | 1.2 |
|  | W3 | 2.2 | 1.6 | 3.0 | 2.2 | 1.5 | 2.4 | 1.4 | 0.4 | 2.4 | 2.6 | 0.9 | 1.7 | 3.1 | 4.0 | 2.5 | 2.0 | 2.2 | 0.9 | 0.8 |
| *Number of …^e^* | |  |  |  |  |  |  |  |  |  |  |  |  |  |  |  |  |  |  |  |
| Bullies | |  |  |  |  |  |  |  |  |  |  |  |  |  |  |  |  |  |  |  |
|  | W1 | 22 | 11 | 19 | 21 | 16 | 12 | 13 | 23 | 11 | 17 | 11 | 16 | 16 | 17 | 15 | 17 | 18 | 17 | 16 |
|  | W2 | 14 | 19 | 17 | 18 | 16 | 16 | 19 | 21 | 17 | 14 | 5 | 20 | 13 | 15 | 15 | 21 | 12 | 16 | 18 |
|  | W3 | 15 | 22 | 19 | 20 | 13 | 11 | 15 | 7 | 18 | 13 | 12 | 20 | 20 | 21 | 16 | 23 | 22 | 12 | 19 |
| Victims | |  |  |  |  |  |  |  |  |  |  |  |  |  |  |  |  |  |  |  |
|  | W1 | 21 | 15 | 14 | 20 | 15 | 8 | 9 | 7 | 13 | 19 | 7 | 8 | 11 | 8 | 8 | 11 | 7 | 9 | 12 |
|  | W2 | 12 | 11 | 10 | 12 | 12 | 10 | 15 | 8 | 11 | 11 | 4 | 11 | 8 | 9 | 8 | 10 | 9 | 10 | 9 |
|  | W3 | 11 | 12 | 8 | 13 | 7 | 14 | 6 | 5 | 10 | 11 | 5 | 7 | 14 | 12 | 12 | 8 | 11 | 4 | 8 |
| *Shared Friendship^f^* | |  |  |  |  |  |  |  |  |  |  |  |  |  |  |  |  |  |  |  |
| Bullying | |  |  |  |  |  |  |  |  |  |  |  |  |  |  |  |  |  |  |  |
|  | W1 | 0.25 | 0.24 | 0.38 | 0.35 | 0.23 | 0.58 | 0.44 | 0.33 | 0.40 | 0.28 | 0.67 | 0.50 | 0.25 | 0.30 | 0.49 | 0.34 | 0.44 | 0.36 | 0.23 |
|  | W2 | 0.44 | 0.31 | 0.32 | 0.34 | 0.37 | 0.35 | 0.35 | 0.33 | 0.55 | 0.38 | 1.00 | 0.34 | 0.37 | 0.26 | 0.50 | 0.32 | 0.50 | 0.48 | 0.40 |
|  | W3 | 0.48 | 0.29 | 0.26 | 0.25 | 0.26 | 0.42 | 0.40 | 0.44 | 0.17 | 0.41 | 0.46 | 0.26 | 0.26 | 0.29 | 0.37 | 0.30 | 0.42 | 0.57 | 0.33 |
| Victimization | |  |  |  |  |  |  |  |  |  |  |  |  |  |  |  |  |  |  |  |
|  | W1 | 0.21 | 0.13 | 0.26 | 0.16 | 0.35 | 0.13 | 0.32 | 0.33 | 0.29 | 0.28 | 0.00 | 0.34 | 0.48 | 0.07 | 0.14 | 0.11 | 0.12 | 0.34 | 0.29 |
|  | W2 | 0.33 | 0.12 | 0.16 | 0.13 | 0.38 | 0.28 | 0.21 | 0.14 | 0.23 | 0.47 | 0.00 | 0.18 | 0.34 | 0.04 | 0.22 | 0.10 | 0.19 | 0.36 | 0.22 |
|  | W3 | 0.24 | 0.26 | 0.36 | 0.05 | 0.18 | 0.26 | 0.17 | 0.08 | 0.04 | 0.31 | 0.06 | 0.10 | 0.22 | 0.13 | 0.26 | 0.19 | 0.16 | 0.05 | 0.13 |
| *Notes.* ^a^Total network size over all waves, including respondents joining, leaving, and staying. ^b^Grade is grade in W1, grade in W2 and W3 is shown in parentheses. ^c^Present (missing) are those who belonged to the classroom and who fill (did not fill) out the questionnaire; Joining are those who were new in the classroom in W2 or W3; Leaving are those who left the classroom in W2 or W3; Staying are those who remained in the classroom in W1 and W2 or W2 and W3. ^d^Average nomination per respondent for friendship or bullying in classroom; ^e^Number of children that sent at least one bully nomination to classmates (bullies) or received at least one bully nomination (victims) from classmates (note that bully nominations were reversed). ^f^Proportion of shared outgoing Y-ties (*i* and *j* bully the same victim *h*) and incoming Y-ties (*i* and *j* are victimized by the same bully *h*) for which there are also outgoing X-ties (*i* and *j* are friends). | | | | | | | | | | | | | | | | | | | | |

| **Table S2** Effects included in all SIENA multivariate network models summarized in Table 2. | | | | | | | | | | | | | | | | | | | |
| --- | --- | --- | --- | --- | --- | --- | --- | --- | --- | --- | --- | --- | --- | --- | --- | --- | --- | --- | --- |
| **Friendship Networks** | 1 | 2 | 3 | 4 | 5 | 6 | 7 | 8 | 9 | 10 | 11 | 12 | 13 | 14 | 15 | 16 | 17 | 18 | 19 |
| *Rate Effects* |  |  |  |  |  |  |  |  |  |  |  |  |  |  |  |  |  |  |  |
| Network rate t1-t2 |  |  |  |  |  |  |  |  |  |  |  |  |  |  |  |  |  |  |  |
| Network rate t2-t3 |  |  |  |  |  |  |  |  |  |  |  |  |  |  |  |  |  |  |  |
| *Structure Effects* |  |  |  |  |  |  |  |  |  |  |  |  |  |  |  |  |  |  |  |
| Outdegree (density) |  |  |  |  |  |  |  |  |  |  |  |  |  |  |  |  |  |  |  |
| Reciprocity |  |  |  |  |  |  |  |  |  |  |  |  |  |  |  |  |  |  |  |
| Indegree popularity |  |  |  |  |  |  |  |  |  |  |  |  |  |  |  |  |  |  |  |
| Outdegree activity |  |  |  |  |  |  |  |  |  |  |  |  |  |  |  |  |  |  |  |
| Transitive triplets |  |  |  |  |  |  |  |  |  |  |  |  |  |  |  |  |  |  |  |
| Transitive reciprocated triplets |  |  |  |  |  |  |  |  |  |  |  |  |  |  |  |  |  |  |  |
| Number of distance 2 |  |  |  |  |  |  |  |  |  |  |  |  |  |  |  |  |  |  |  |
| Four-cycles |  |  |  |  |  |  |  |  |  |  |  |  |  |  |  |  |  |  |  |
| *Sex Effects* |  |  |  |  |  |  |  |  |  |  |  |  |  |  |  |  |  |  |  |
| Same sex |  |  |  |  |  |  |  |  |  |  |  |  |  |  |  |  |  |  |  |
| *Dyadic Multiplex Effects* |  |  |  |  |  |  |  |  |  |  |  |  |  |  |  |  |  |  |  |
| Existing tie W → new tie X |  |  |  |  |  |  |  |  |  |  |  |  |  |  |  |  |  |  |  |
| *Degree-Related Multiplex Effects* |  |  |  |  |  |  |  |  |  |  |  |  |  |  |  |  |  |  |  |
| Indegree tie W → indegree tie X |  |  |  |  |  |  |  |  |  |  |  |  |  |  |  |  |  |  |  |
| Outdegree tie W → indegree tie X |  |  |  |  |  |  |  |  |  |  |  |  |  |  |  |  |  |  |  |
| Outdegree tie W → outdegree tie X |  |  |  |  |  |  |  |  |  |  |  |  |  |  |  |  |  |  |  |
| *Mixed Triadic Multiplex Effects* |  |  |  |  |  |  |  |  |  |  |  |  |  |  |  |  |  |  |  |
| From tie W → agreement tie X |  |  |  |  |  |  |  |  |  |  |  |  |  |  |  |  |  |  |  |
| Tie W → to agreement tie X |  |  |  |  |  |  |  |  |  |  |  |  |  |  |  |  |  |  |  |
| *Fit Improvement Effects* |  |  |  |  |  |  |  |  |  |  |  |  |  |  |  |  |  |  |  |
| Outdegree at least 1 (isolates) |  |  |  |  |  |  |  |  |  |  |  |  |  |  |  |  |  |  |  |
| Outdegree at least 3 |  |  |  |  |  |  |  |  |  |  |  |  |  |  |  |  |  |  |  |
| Outdegree at least 4 |  |  |  |  |  |  |  |  |  |  |  |  |  |  |  |  |  |  |  |
| Structural equivalence indegree |  |  |  |  |  |  |  |  |  |  |  |  |  |  |  |  |  |  |  |
| Structural equivalence outdegree |  |  |  |  |  |  |  |  |  |  |  |  |  |  |  |  |  |  |  |
| **Bullying Networks** | 1 | 2 | 3 | 4 | 5 | 6 | 7 | 8 | 9 | 10 | 11 | 12 | 13 | 14 | 15 | 16 | 17 | 18 | 19 |
| *Rate Effects* |  |  |  |  |  |  |  |  |  |  |  |  |  |  |  |  |  |  |  |
| Network rate t1-t2 |  |  |  |  |  |  |  |  |  |  |  |  |  |  |  |  |  |  |  |
| Network rate t2-t3 |  |  |  |  |  |  |  |  |  |  |  |  |  |  |  |  |  |  |  |
| *Structure Effects* |  |  |  |  |  |  |  |  |  |  |  |  |  |  |  |  |  |  |  |
| Outdegree (density) |  |  |  |  |  |  |  |  |  |  |  |  |  |  |  |  |  |  |  |
| Outdegree at least 1 (isolates) |  |  |  |  |  |  |  |  |  |  |  |  |  |  |  |  |  |  |  |
| Indegree at least 1 (isolates) |  |  |  |  |  |  |  |  |  |  |  |  |  |  |  |  |  |  |  |
| Reciprocity |  |  |  |  |  |  |  |  |  |  |  |  |  |  |  |  |  |  |  |
| Indegree popularity |  |  |  |  |  |  |  |  |  |  |  |  |  |  |  |  |  |  |  |
| Outdegree activity |  |  |  |  |  |  |  |  |  |  |  |  |  |  |  |  |  |  |  |
| Transitive triplets |  |  |  |  |  |  |  |  |  |  |  |  |  |  |  |  |  |  |  |
| Number of distance 2 |  |  |  |  |  |  |  |  |  |  |  |  |  |  |  |  |  |  |  |
| Four-cycles |  |  |  |  |  |  |  |  |  |  |  |  |  |  |  |  |  |  |  |
| *Sex Effects* |  |  |  |  |  |  |  |  |  |  |  |  |  |  |  |  |  |  |  |
| Same sex |  |  |  |  |  |  |  |  |  |  |  |  |  |  |  |  |  |  |  |
| *Dyadic Multiplex Effects* |  |  |  |  |  |  |  |  |  |  |  |  |  |  |  |  |  |  |  |
| Existing tie W → new tie X |  |  |  |  |  |  |  |  |  |  |  |  |  |  |  |  |  |  |  |
| *Degree-Related Multiplex Effects* |  |  |  |  |  |  |  |  |  |  |  |  |  |  |  |  |  |  |  |
| Indegree tie W → indegree tie X |  |  |  |  |  |  |  |  |  |  |  |  |  |  |  |  |  |  |  |
| Outdegree tie W → indegree tie X |  |  |  |  |  |  |  |  |  |  |  |  |  |  |  |  |  |  |  |
| Outdegree tie W → outdegree tie X |  |  |  |  |  |  |  |  |  |  |  |  |  |  |  |  |  |  |  |
| *Mixed Triadic Multiplex Effects* |  |  |  |  |  |  |  |  |  |  |  |  |  |  |  |  |  |  |  |
| From tie W → agreement tie X |  |  |  |  |  |  |  |  |  |  |  |  |  |  |  |  |  |  |  |
| Tie W → closure agreement tie X |  |  |  |  |  |  |  |  |  |  |  |  |  |  |  |  |  |  |  |
| *Notes.* All models were analyzed using 10,000 iterations for better convergence and reliability of the parameter estimates and standard errors. In some classrooms, the rate effects for bullying became unreasonably high (e.g., larger than 20). As a possible solution, the rate effects were fixed at the observed value (Ripley et al., 2019). Other effects were fixed at a non-zero value (based on score-type tests) to obtain convergence when the networks did not carry enough information to estimate them; otherwise effects were fixed to zero (when a non-zero value resulted in non-convergence). Fixed parameters are indicated with yellow cells. White cells indicate effects *not* included for model fit (only for ‘*Fit Improvement Effects*’). | | | | | | | | | | | | | | | | | | | |

| **Table S3** Goodness of Fit statistics for SIENA multivariate network models summarized in Table 2. | | | | | | | | | |
| --- | --- | --- | --- | --- | --- | --- | --- | --- | --- |
|  | | Friendship Networks | | | | Bullying Networks | | | |
| Classroom | Overall convergence | Outdegree distribution | Indegree distribution | Geodesic distance | Triad census | Outdegree distribution | Indegree distribution | Geodesic distance | Triad census |
| 1 | 0.15 | 0.71 | 0.06 | 0.60 | 0.05 | 0.55 | 0.98 | 0.97 | 0.62 |
| 2 | 0.14 | 0.20 | 0.82 | 0.37 | 0.22 | 0.64 | 1 | 0.93 | 0.97 |
| 3 | 0.18 | 0.11 | 0.92 | 0.97 | 0.14 | 0.12 | 0.98 | 0.01 | 0.81 |
| 4 | 0.16 | 0.26 | 0.41 | 0.91 | 0.14 | 0.72 | 0.13 | 0.85 | 0.10 |
| 5 | 0.14 | 0.14 | 0.30 | 0.96 | 0.67 | 0.98 | 0.76 | 0.89 | 0.76 |
| 6 | 0.15 | 0.31 | 0.87 | 0.81 | 0.08 | 0.26 | 0.81 | 0.94 | 0.75 |
| 7 | 0.15 | 0.84 | 0.81 | 0.34 | 0.79 | 0.85 | 0.96 | 0.59 | 0.57 |
| 8 | 0.16 | 0.97 | 0.22 | 0.63 | 0.64 | 0.92 | 0.52 | 0.32 | 0.57 |
| 9 | 0.14 | 0.60 | 0.82 | 0.34 | 0.06 | 0.99 | 0.96 | 0.76 | 0.60 |
| 10 | 0.17 | 0.52 | 0.67 | 0.99 | 0.19 | 0.87 | 0.69 | 1 | 0.60 |
| 11 | 0.15 | 0.45 | 0.56 | 0.96 | 0.71 | 0.96 | 0.45 | 0.95 | 0.81 |
| 12 | 0.20 | 0.29 | 0.001 | 0.38 | 0.18 | 0.44 | 0.01 | 0.49 | 0.86 |
| 13 | 0.17 | 0.34 | 0.92 | 0.25 | 0.004 | 0.65 | 0.59 | 0.84 | 0.37 |
| 14 | 0.13 | 0.06 | 0.08 | 0.91 | 0.39 | 0.61 | 0.02 | 0.98 | 0.05 |
| 15 | 0.16 | 0.22 | 0.85 | 0.99 | 0.85 | 0.92 | 0.79 | 0.91 | 0.99 |
| 16 | 0.14 | 0.76 | 0.49 | 0.79 | 0.83 | 0.75 | 0.22 | 0.88 | 0.26 |
| 17 | 0.19 | 0.31 | 0.47 | 0.30 | 0.004 | 0.33 | 0.46 | 0.92 | 0.80 |
| 18 | 0.14 | 0.14 | 0.41 | 1 | 0.77 | 0.50 | 0.90 | 0.80 | 0.99 |
| 19 | 0.19 | 0.73 | 0.19 | 0.61 | 0.53 | 0.73 | 0.65 | 0.36 | 0.90 |
| *Notes*. Goodness of fit (GoF) was assessed with four computed auxiliary network statistics: outdegree distribution, indegree distribution, geodesic distance, and triad census. The auxiliary network statistics are important indices for how well friendship or bully-victim patterns in the classroom network are represented with the included model effects or whether additional effects are needed. For each auxiliary statistic, the differences between the values in the observed classroom network (summed across the three waves of data) and the simulated values in the model (summed across 1,000 random networks) are assessed with the Mahalonobis distance (cf. Ripley et al., 2019). Fit for a particular statistic is good or acceptable when the reported *p*-value is larger than .05, indicating that the simulated values do not depart too much from the observed values. This is illustrated in Figure S1 with violin plots. These violin plots can be used to inspect whether, for a specific statistic, there are too ‘many’ (or too ‘few’ or a ‘sufficient’ number of) values being simulated in comparison to the observed values with a five percent margin of error. The red solid lines shows the observed values; the boxplots and violins show the distribution of the simulated values. There are several classrooms for which one or more statistics are not well fitted. These are indicated with yellow cells. In two classrooms (11 and 15), additional effects included improved fit for Outdegree distribution and Triad census for friendship (see Table S1 for the complete list of effects). Five other classrooms (classroom 3: geodesic distance for bullying; classroom 12: indegree distribution for friendship and bullying; classroom 13: triad census for friendship; classroom 14: indegree distribution and triad census for bullying; and classroom 17: triad census for friendship), fit was not optimal for one or more statistics. This can also be seen in Figure S1. Closer inspection reveals that the model overrepresented the number of indirect bullying relationships for classroom 3. In classroom 12, a few children received a disproportionate amount of friendship nominations at the first moment, but not at later moments; in addition, a non-victimized child at wave 1 was bullied by many classmates later on. In classroom 14, some children were bullied more over time, whereas others were bullied less. A specific triadic configuration was not well represented for friendship in classroom 13 (structural equivalence regarding incoming nominations for friendship) and 17 (indirect connections through one intermediary). Our included parameters did not capture such configurations well. Unfortunately, adding additional effects did not contribute to a better model fit and were therefore not included. | | | | | | | | | |

| **Figure S1** Visualization of Goodness of Fit statistics of SIENA multivariate network models summarized in Table 2. | | | |
| --- | --- | --- | --- |
| **Friendship Networks** (Classroom 1 to 6) | | | |
| Outdegree distribution | Indegree distribution | Geodesic distribution | Triad census |
|  |  |  |  |
|  |  |  |  |
|  |  |  |  |
|  |  |  |  |
|  |  |  |  |
|  |  |  |  |
| **Figure S1** *Continues on next page.* | | | |

| **Figure S1** *Continued.* | | | |
| --- | --- | --- | --- |
| **Friendship Networks** (Classroom 7 to 12) | | | |
| Outdegree distribution | Indegree distribution | Geodesic distribution | Triad census |
|  |  |  |  |
|  |  |  |  |
|  |  |  |  |
|  |  |  |  |
|  |  |  |  |
|  |  |  |  |
| **Figure S1** *Continues on next page.* | | | |

| **Figure S1** *Continued.* | | | |
| --- | --- | --- | --- |
| **Friendship Networks** (Classroom 13 to 18) | | | |
| Outdegree distribution | Indegree distribution | Geodesic distribution | Triad census |
|  |  |  |  |
|  |  |  |  |
|  |  |  |  |
|  |  |  |  |
|  |  |  |  |
|  |  |  |  |
| **Figure S1** *Continues on next page.* | | | |

| **Figure S1** *Continued.* | | | |
| --- | --- | --- | --- |
| **Friendship Networks** (Classroom 19) | | | |
| Outdegree distribution | Indegree distribution | Geodesic distribution | Triad census |
|  |  |  |  |
| **Bullying Networks** (Classroom 1 to 5) | | | |
|  |  |  |  |
|  |  |  |  |
|  |  |  |  |
|  |  |  |  |
|  |  |  |  |
| **Figure S1** *Continues on next page.* | | | |

| **Figure S1** *Continued.* | | | |
| --- | --- | --- | --- |
| **Bullying Networks** (Classroom 6 to 11) | | | |
| Outdegree distribution | Indegree distribution | Geodesic distribution | Triad census |
|  |  |  |  |
|  |  |  |  |
|  |  |  |  |
|  |  |  |  |
|  |  |  |  |
|  |  |  |  |
| **Figure S1** *Continues on next page.* | | | |

| **Figure S1** *Continued.* | | | |
| --- | --- | --- | --- |
| **Bullying Networks** (Classroom 12 to 17) | | | |
| Outdegree distribution | Indegree distribution | Geodesic distribution | Triad census |
|  |  |  |  |
|  |  |  |  |
|  |  |  |  |
|  |  |  |  |
|  |  |  |  |
|  |  |  |  |
| **Figure S1** *Continues on next page.* | | | |

| **Figure S1** *Continued.* | | | |
| --- | --- | --- | --- |
| **Bullying Networks** (Classroom 18 to 19) | | | |
| Outdegree distribution | Indegree distribution | Geodesic distribution | Triad census |
|  |  |  |  |
|  |  |  |  |

| **Figure S2** Distribution of parameter estimates and standard deviations for SIENA multivariate network models summarized in Table 2. | | | | | |
| --- | --- | --- | --- | --- | --- |
| **Friendship Networks** | | | **Bullying Networks** | | |
|  |  |  |  |  |  |
|  |  |  |  |  |  |
|  |  |  |  |  |  |
|  |  |  |  |  |  |
|  |  |  |  |  |  |
|  |  |  |  |  |  |

| **Table S4** Results of time heterogeneity tests of selection effects and influence effects in bullying and victimization. | | | | | | | | | | | |
| --- | --- | --- | --- | --- | --- | --- | --- | --- | --- | --- | --- |
| Classroom | | 1 | 2 | 3 | 4 | 5 | 6 | 7 | 8 | 9 | 10 |
| **Bullying** | |  |  |  |  |  |  |  |  |  |  |
|  | Selection | 0.76+ | 0.49+ | 3.30+ | 4.52− | 0.82− | 3.20− | 7.63− | 0.84+ | 0.52+ | 0.19+ |
|  | Influence | 3.14− | 0.20+ | 0.07+ | 4.51+ | 0.51− | 8.06+ | 73.27+ | 3.22− | 1.20+ | 0.47+ |
| **Victimization** | |  |  |  |  |  |  |  |  |  |  |
|  | Selection | 4.44− | 1.85− | 2.71+ | 3.74− | 0.11+ | 0.63+ | 0.28− | 0.01+ | 4.30+ | 9.53− |
|  | Influence | 5.70+ | 0.82− | 0.00+ | 10.54− | 0.17+ | 20.77− | 164.60+ | 2.19− | 0.14+ | 0.01− |
| Classroom | | 11 | 12 | 13 | 14 | 15 | 16 | 17 | 18 | 19 |  |
| **Bullying** | |  |  |  |  |  |  |  |  |  |  |
|  | Selection | 4.50+ | 0.39− | 4.02− | 0.03+ | 0.00+ | 1.07+ | 1.90+ | 0.01+ | 0.48+ |  |
|  | Influence | 6.88+ | 2.02+ | 0.61− | 0.58− | 6.37+ | 3.82− | 28.57+ | 2.04− | NA |  |
| **Victimization** | |  |  |  |  |  |  |  |  |  |  |
|  | Selection | 0.01+ | 1.30− | 4.59− | 0.19− | NA | 0.10+ | 0.86− | 0.18− | 11.06− |  |
|  | Influence | 0.76− | NA | 3.88+ | 1.35+ | 16.24+ | 0.74− | 0.02− | 8.95− | 2.07+ |  |
| *Notes.* *Differences in effect parameters (as reported in Table 2 in the Manuscript) between estimation periods (W1-W2 vs. W2-W3) assessed with chi-square test using sienaTimeTest (see RSiena manual; Ripley et al., 2019); Significant differences shown in red; Sign shows direction of differences (+ more positive in W2-W3, – more negative in W2-W3). Bullying selection assessed with the “from” effect in RSiena (agreement along W leading to X, where W is an outgoing bullying tie and X is an outgoing friendship tie); bullying influence assessed with the “to” effect in RSiena (W leading to agreement along X, where W is an outgoing friendship tie and X is outgoing bullying tie); victimization selection assessed with the “sharedIn” effect in RSiena (closure of shared incoming WW → X, where W is an incoming bullying tie and X is outgoing friendship tie); victimization influence assessed with the “cl.XWX” effect in RSiena (XWX closure of W, where W is an outgoing friendship tie and X is an outgoing bullying tie). | | | | | | | | | | | |

| **Table S5** Results from longitudinal multivariate network models predicting co-evolution of friendship and bullying (14 classrooms, 352 students) – excluding the five classrooms with no optimal fit (3, 12, 13, 14, 17). | | | | | | | | | | | | |
| --- | --- | --- | --- | --- | --- | --- | --- | --- | --- | --- | --- | --- |
|  |  |  |  | | |  | **Friendship Networks** | | | **Bullying Networks** | | |
|  |  |  | Hypothetical change | | |  |  | | |  | | |
|  |  |  | t*x* | → | t*x* + *m* |  | Est. | SE | *n* | Est. | SE | *n* |
| Effect Parameters | |  |  |  |  |  |  |  |  |  |  |  |
| *Rate Effects* | |  |  |  |  |  |  |  |  |  |  |  |
|  | Network rate t1→t2 |  |  |  |  |  | 8.21*** | 0.58^a^ | 14 | 7.60*** | 1.35^a^ | 10 |
|  | Network rate t2→t3 |  |  |  |  |  | 7.90*** | 0.57^a^ | 14 | 6.06*** | 0.49 | 10 |
| *Structure Effects* | |  |  |  |  |  |  |  |  |  |  |  |
|  | Outdegree (density) |  |  | → |  |  | -2.46*** | 0.24 | 14 | -1.98*** | 0.25 | 14 |
|  | Outdegree isolates |  |  | → |  |  |  |  |  | -1.46*** | 0.36^a^ | 14 |
|  | Indegree isolates |  |  | → |  |  |  |  |  | -2.22*** | 0.28 | 13 |
|  | Reciprocity |  |  | → |  |  | 1.60*** | 0.16 | 14 | 0.38+ | 0.22 | 13 |
|  | Indegree popularity |  |  | → |  |  | 0.07 | 0.07 | 14 | -0.008 | 0.09 | 14 |
|  | Outdegree activity |  |  | → |  |  | 0.02 | 0.05 | 14 | 0.09 | 0.09 | 14 |
|  | Transitive triplets |  |  | → |  |  | 0.25** | 0.09 | 14 | 0.04 | 0.15 | 14 |
|  | Transitive reciprocated triplets |  |  | → |  |  | -0.32** | 0.11 | 14 |  |  |  |
|  | Actors at distance 2 |  |  | → |  |  | -0.23** | 0.08 | 14 | -0.28+ | 0.16 | 14 |
|  | Four-cycles |  |  | → |  |  | -0.03 | 0.04 | 14 | -0.06 | 0.07 | 14 |
| *Sex Effects* | |  |  |  |  |  |  |  |  |  |  |  |
|  | Same sex |  |  | → |  |  | 0.63*** | 0.14 | 14 | 0.01 | 0.16 | 14 |
| *Dyadic Multiplex Effects* | |  |  |  |  |  |  |  |  |  |  |  |
|  | Existing tie W → new tie X |  |  | → |  |  | -0.35 | 0.38 | 13 | -0.06 | 0.30 | 13 |
| *Degree-Related Multiplex Effects* | |  |  |  |  |  |  |  |  |  |  |  |
|  | Indegree tie W → Indegree tie X |  |  | → |  |  | -0.006 | 0.07 | 14 | -0.02 | 0.07 | 14 |
|  | Outdegree tie W → Indegree tie X |  |  | → |  |  | -0.12 | 0.11 | 14 | 0.005 | 0.11 | 14 |
|  | Outdegree tie W → Outdegree tie X |  |  | → |  |  | -0.01 | 0.10 | 13 | -0.15+ | 0.09 | 14 |
| *Mixed Triadic Multiplex Effects^b^* | |  |  |  |  |  |  |  |  |  |  |  |
|  | H1: shared victim to friendship |  |  | → |  |  | 0.42* | 0.18 | 14 |  |  |  |
|  | H2: friendship agreement to bullying |  |  | → |  |  |  |  |  | 0.74*** | 0.16 | 14 |
|  | H3: shared bully to friendship |  |  | → |  |  | 0.06 | 0.11 | 13 |  |  |  |
|  | H4: friendship agreement to victimization |  |  | → |  |  |  |  |  | -0.02 | 0.14 | 13 |
| *Notes.* Significance tests performed by dividing the estimates with its standard error resulting in *t*-Values which under the null hypothesis are approximately normally distributed (Ripley et al., 2019); +*p* ≤ .10, **p* ≤ .05, **p* ≤ .01, **p* ≤ .001 (two-tailed test). Convergence statistics: t ratios all < 0.07; Overall maximum convergence ratio < 0.21. ^a^Significant differences between classrooms. ^b^To facilitate the interpretation of these network configurations, friendships are represented with solid lines and bullying relationships are represented with dashed lines. | | | | | | | | | | | | |
